# Saline Infusion

**Published:** 1908-05-30

**Authors:** 


### SALINE INFUSION.

Infusion of saline solution into the subcutaneous tissues of the axilla or of the legs, or into a vein, or into the rectum, is a measure of undoubted usefulness in certain cases, notably in conditions of collapse from hæmorrhage or from peritonitis, or in the shrunken condition of infants suffering from diarrhœa and vomiting; the main indication for infusion is that the patient's tissues should have been in some way depleted of their full quantum of water, and it is now held to be very preferable to replace that water by slow, continuous infusion rather than by the rapid injection of a large quantity in a short time.

It is not so much, however, of the methods and benefits of infusion that we would speak here, but rather of the nature of the fluid with which infusion should be performed. The one and only object kept in mind, as a rule, has been to ensure that this fluid should be isotonic with the blood—that is to say, that when blood is mixed with the infusion fluid the proportion of salts in the latter shall be exactly such that no water should pass from it into the red corpuscles nor any be drawn from the red corpuscles into the fluid. We venture to think that far too little stress has hitherto been laid upon the nature of the salts used to make the fluid isotonic with the blood.

Ordinary normal saline solution is made by adding rather more than a drachm of sodium chloride to each pint of water. This, it is true, is isotonic with the blood, but it has long been known to physiologists, since Ringer's experiments were made upon the perfusion of frogs' hearts, that a solution of sodium chloride alone is far less efficacious in stimulating the heart to its best contractions than is an isotonic solution containing other salts besides—notably those of calcium and potassium. It is surprising that this physiological fact has not been fully brought to bear in the treatment of human beings. Ringer's solution, made isotonic for human blood, would be a far better liquid for infusing with than is normal saline.

The following is a liquid which has been recommended for the purpose:—

| | |
|---|---|
| ℞ Sodii chloridi ... ... ... | 6.50 grm. |
| Potassii chloridi ... ... ... | 0.30 grm. |
| Calcii chloridi ... ... ... | 0.20 grm. |
| Magnesii sulphatis ... ... ... | 0.30 grm. |
| Sodii bicarbonatis ... ... ... | 1.00 grm. |
| Sodii glycerophosphatis ... ... | 1.00 grm. |
| Glucosi ... ... ... ... | 1.00 grm. |
| Aquam destillatam ad ... | 1000.00 c.c. |
| Oxygen gas to saturation. | |

The oxygen is added with the idea of making the fluid still more like the blood plasma; the glucose is not essential, but it is held to be of service. The proportions of the other ingredients need not be altered if the glucose is left out.

This solution has the power of keeping spermatozoa alive for several days if the temperature is maintained at that of the body. Spermatozoa will not live in this way in ordinary normal saline, which is a good argument in favour of the less simple fluid for purposes of transfusion or infusion. It has, indeed, been used in many cases on the Continent, and its advantages have been demonstrated to be not merely theoretical.

